# Evolution of white matter hyperintensity segmentation methods and implementation over the past two decades; an incomplete shift towards deep learning

**DOI:** 10.1007/s11682-024-00902-w

**Published:** 2024-07-31

**Authors:** Maryam Rahmani, Donna Dierker, Lauren Yaeger, Andrew Saykin, Patrick H. Luckett, Andrei G. Vlassenko, Christopher Owens, Hussain Jafri, Kyle Womack, Jurgen Fripp, Ying Xia, Duygu Tosun, Tammie L. S. Benzinger, Colin L. Masters, Jin-Moo Lee, John C. Morris, Manu S. Goyal, Jeremy F. Strain, for the ADOPIC, ADNI Investigators, Walter Kukull, Michael Weiner, Biostats, Database and Bioinformatics, Samantha Burnham, Tim James CoxDoecke, Victor Fedyashov, Jurgen Fripp, Rosita Shishegar, Chengjie Xiong, Daniel Marcus, Parnesh Raniga, Shenpeng Li, Cognition, Andrew Aschenbrenner, Jason Hassenstab, Yen Ying Lim, Paul Maruff, Hamid Sohrabi, Jo Robertson, Shaun Markovic, Imaging, Pierrick Bourgeat, Vincent Doré, Clifford Jack Mayo, Parinaz Mussoumzadeh, Chris Rowe, Victor Villemagne, CSF and Blood, Randy Bateman, Chris Fowler, Qiao-Xin Li, Ralph Martins, Suzanne Schindler, Les Shaw, Genetics, Carlos Cruchaga, Oscar Harari, Simon Laws, Tenielle Porter, Eleanor O’Brien, Neuropathology, Richard Perrin, NACC, Walter Kukull, DIAN, Randy Bateman, Eric McDade, Cerebrovascular Disease (CVD) Risk, Clifford Jack, John Morris, Nawaf Yassi, Hippocampal Sclerosis (HS-TDP43) Risk, Pierrick Bourgeat, Richard Perrin, Blaine Roberts, Victor Villemagne, Artificial Intelligence and Machine Learning, Victor Fedyashov, Benjamin Goudey

**Affiliations:** 1 Department of Neurology, Washington University School of Medicine, St. Louis, MO USA; 2 Mallinckrodt Institute of Radiology, Washington University School of Medicine, St. Louis, MO USA; 3 Knight Alzheimer Disease Research Center, St. Louis, MO USA; 4 Department of Biomedical Engineering, Washington University in St. Louis, St. Louis, MO USA; 5 Division of Radiology and Biomedical Imaging, University of CA – San Francisco, San Francisco, CA USA; 6 The Australian E-Health Research Centre, CSIRO Health and Biosecurity, Brisbane, QLD Australia; 7 The Florey Institute of Neuroscience and Mental Health, The University of Melbourne, Parkville, VIC Australia; 8 Neuroimaging Labs Research Center, Washington University School of Medicine, St. Louis, MO USA; 9 Department School of Medicine, Indiana University, Bloomington, IN USA; 10 Division of Neurotechnology, Department of Neurological Surgery, Washington University School of Medicine, St. Louis, MO USA

**Keywords:** White matter hyperintensity, Segmentation, Pipelines, Deep Learning, Systematic Review

## Abstract

This systematic review examines the prevalence, underlying mechanisms, cohort characteristics, evaluation criteria, and cohort types in white matter hyperintensity (WMH) pipeline and implementation literature spanning the last two decades. Following Preferred Reporting Items for Systematic Reviews and Meta-Analysis (PRISMA) guidelines, we categorized WMH segmentation tools based on their methodologies from January 1, 2000, to November 18, 2022. Inclusion criteria involved articles using openly available techniques with detailed descriptions, focusing on WMH as a primary outcome. Our analysis identified 1007 visual rating scales, 118 pipeline development articles, and 509 implementation articles. These studies predominantly explored aging, dementia, psychiatric disorders, and small vessel disease, with aging and dementia being the most prevalent cohorts. Deep learning emerged as the most frequently developed segmentation technique, indicative of a heightened scrutiny in new technique development over the past two decades. We illustrate observed patterns and discrepancies between published and implemented WMH techniques. Despite increasingly sophisticated quantitative segmentation options, visual rating scales persist, with the SPM technique being the most utilized among quantitative methods and potentially serving as a reference standard for newer techniques. Our findings highlight the need for future standards in WMH segmentation, and we provide recommendations based on these observations.

**Supplementary Information:**

The online version contains supplementary material available at 10.1007/s11682-024-00902-w.

## Introduction

White matter hyperintensities (WMH) are a unique MRI signature present in various pathologies, including typical aging (Georgakis et al., 2019; Morris et al., 2009). WMH are defined as abnormally high-intensity regions within the white matter on T2-weighted fluid attenuated imaging recovery (FLAIR) images. Traditionally, WMH were visually assessed using different rating scales or manually delineated to quantify a continuous estimate of lesion severity (Scheltens et al., 1998). However, both of these approaches have substantial limitations, and therefore, more automated approaches have emerged for quantifying WMH burden (Heuvel et al., 2006). Past reviews on WMH techniques focused on comparing and contrasting specific techniques and overlook their contribution to the field (Kuijf et al., 2019; Li et al., 2022), (Balakrishnan et al., 2021). In this review, we take a novel historical approach and qualitatively determine patterns in the cohort, cohort size, and segmentation methodology. These categories are evaluated independently in both WMH pipeline development articles (i.e., those that define the technique of a given segmentation method) and implementation articles (i.e., those that evaluate WMH as a primary variable of interest) over the past two decades.

Maximizing the reliability and robustness of lesion segmentation has long been the overarching goal in the field and has led to a variety of different techniques (Kuijf et al., 2019). At present, no specific methodology is universally accepted as a reference standard; thus, there is a high degree of heterogeneity in the techniques used (Melazzini et al., 2021). Not only do segmentation techniques differ between studies, but the evaluation process of novel segmentation methodologies is also inconsistent due to the absence of a reference standard. Manual segmentations are often used as a reference standard; however, manual methods can introduce rater bias, vary significantly between studies, and are vulnerable to differences in FLAIR acquisition methods. Moreover, even if a reference standard was available, there are no set standards or guidelines for evaluating new WMH pipelines, which are typically established at the authors' discretion. This lack of consensus on evaluation guidelines may further contribute to conflicting findings in published studies and clinical trials.

Yet another source of variability in WMH studies is that they can reflect different biological properties based on different pathologies. WMH occur in aging (Li et al., 2023), neurodegenerative diseases (Dadar et al., 2020), cardiovascular incidents (Arai & Arai, 2023), multiple sclerosis (MS) (Paolini Paoletti et al., 2021), and numerous other pathologies. It remains unclear whether methods used to segment WMH in one disease state are valid for another disease. Even within the same individuals of a cohort, such as those studying aging and Alzheimer disease, the pathogenesis of WMH might vary. Given the importance of WMH to a variety of prevalent neurological conditions, there is broad interest in the development of better WMH segmentation methods. This includes multiple research-focused and commercial techniques, some of which are now being incorporated into clinical practice and trials (Brown et al., 2021). Thus, investigating the evolution of WMH segmentation methodology provides an unique perspective on how advances in computational methods might impact neuroimaging, with implications for both research and clinical practice.

Accordingly, this review aims to comprehensively evaluate a broad range of WMH articles over the prior two decades to produce a historical perspective on the development and implementation of segmentation methodologies. We categorize pipeline and implementation articles based on various features, including segmentation methodology, cohort, cohort size, validation or efficacy assessment approaches, and neuroimaging databases utilized. Our goal is to outline how the field has developed to date and provide a vantage point for future efforts that might help create a more uniform approach to WMH segmentation.

## Methods

**Fig. 1:**
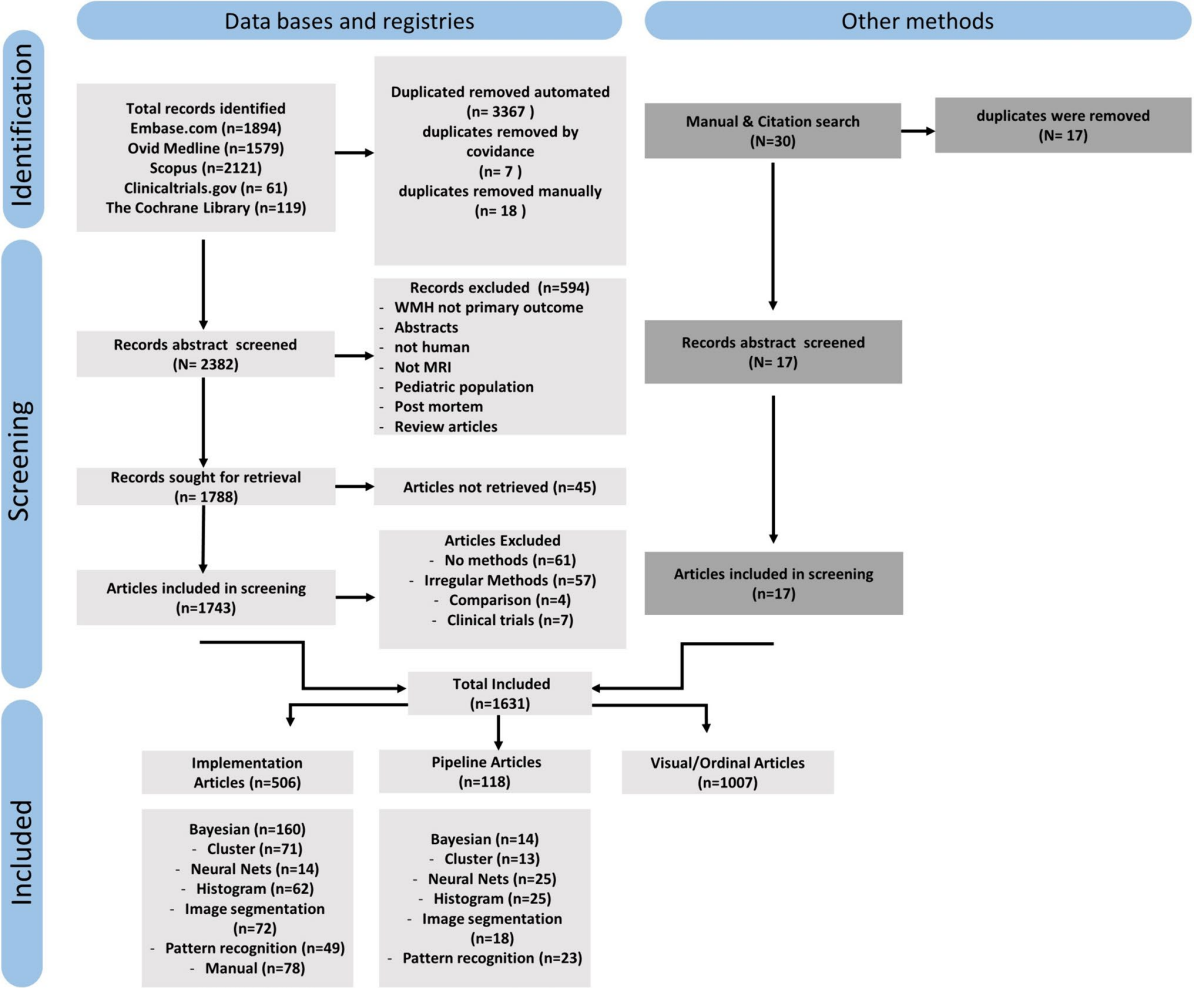
PRISMA outline chart for the selection of manuscripts included into this review

Search strategies and article screening were done using the Preferred Reporting Items for Systematic Reviews and Meta-Analyses (PRISMA) guidelines. A medical librarian (LY) created keywords and a controlled vocabulary based on variation of terms used in the literature to describe WMH according to Standards for reporting vascular changes on neuroimaging (STRIVE) 2013 guidelines(Wardlaw et al., 2013). Further, keywords were added to cover all designated segmentation strategies and, importantly, non-quantifying keywords to cover visual scales (i.e., burden). Articles were acquired across five databases, including Embase.com (n = 1894), Ovid Medline (n = 1579), Scopus (n = 2121), Clinicaltrials.gov (n = 61), and Cochrane Central Register of Controlled Trials (CENTRAL) (n = 119). No specific search term was included to identify articles covering MS lesions. All search strategies were updated on November 18, 2022. Inclusion criteria included articles 1) published between 2000–2022, 2) in the English language, and 3) describing studies of adult participants; our search delivered 5,274 observations. We included pipeline articles trained on MS cohorts, but MS implementation articles were not included. 3,367 duplicate records were deleted after using the de-duplication processes described in “De-duplication of database search results for systematic reviews in EndNote,” (Bramer et al., 2016) another seven records were removed after being screened with the database tool Covidence, and 18 were manually removed resulting in a total of 2,382 unique citations included in the project library, An additional 30 articles were detected based on missing pipeline articles identified within the citations of implementation articles that survived our search criteria form which an additional 17 unique articles were included (Fig. 1). Fully reproducible search strategies and queries for each database can be found in the appendix.

Abstracts and full articles were then manually screened by JS and MR based on the exclusion criteria 1) no access to the full article, 2) non-MRI imaging modalities, 3) non-adult, 4) non-human, 5) postmortem, 6) review articles that didn’t introduce a new technique, 7) articles where WMH was not designated as a primary outcome, and 8) if the methods were poorly described without citations or based on proprietary or in-house methods. 768 manuscripts were removed based on the aforementioned criteria, yielding 1631 manuscripts that met all inclusion and exclusion criteria. All papers were reviewed in Covidence and exported as spreadsheets into Matlab for figure visualization. Due to the scope of this review, we did not include these selected papers in the present reference section unless specifically mentioned; instead, a full list of all papers included in this review can be found in the supplemental Table 1.

### Data extraction

Each article that survived our inclusion/exclusion criteria was initially classified into three broad categories: visual, pipeline development, or segmentation article. Manuscripts were defined as visual if no quantitative measures were used, and all assessments of WMH severity were based on visual rating scales. Pipeline development articles were defined if the author(s) introduced a new segmentation tool or no prior methods were referenced. Segmentation articles represented manuscripts that utilized segmentation tools for quantifying continuous measures for WMH severity but did not discuss or originally develop the segmentation method. No pipeline article was included in the evaluation of segmentation articles to ensure each publication only contributed to their assigned group.

### Segmentation type

The manuscripts defined as visual were not subdivided further based on the type of visual scale, but additional subcategories for the segmentation or pipeline papers were devised to better identify patterns of scientific evaluation over the past two decades. Based on previously published classification schemes (Caligiuri et al., 2015), (Wang et al., 2015), (Balakrishnan et al., 2021), (Qin et al., 2018), (Lee et al., 2015), we created a hierarchical 7-class classification system (segmentation type categories) that best-identified types of segmentation methodology for WMH quantification. 1) Manual-based approaches were defined by single or multiple raters delineating lesion voxels from non-lesion voxels with no computer assistance. 2) Intensity-based thresholding approaches consisted of methodologies that derived an arbitrary or tested threshold to define likely lesion versus not lesion, which often included manual intervention to remove false positives. 3) Histogram approaches devised a Gaussian distribution on the tested or third-party cohort to determine lesion delineation based on the intensity extremes. 4) Clustering methods utilized unsupervised approaches that did not require training or fine-tuning to determine WMH boundaries. 5) Bayesian regression models can include FLAIR, T1, and/or other imaging modalities to create a voxel-wise prediction on the likelihood of tissue type, including WMH. 6) Pattern classification involved supervised methods to fine-tune algorithms to best identify WMH clusters. 7) Deep learning (DL) models encompass supervised, unsupervised, and semi-supervised approaches and included various types of deep learning models/architectures (e.g., convolutional neural networks, U-Net architectures, generative adversarial networks) and methodologies (e.g., data set, training/validation criteria, modality used for input, etc.) for automated segmentation of WMH regions. Any manuscript that incorporated a hybrid approach was classified to the numerically highest classification group to avoid any article from contributing more than once to the full analysis.

### Trends and patterns evaluated

All publications that used WMH as a primary outcome variable were evaluated to determine if, at any year over the past two decades, an increase in WMH publications was observed. Additionally, data regarding sample size, participant databases, cohort, and segmentation type were extracted from each article. The median sample size was calculated for each segmentation type across pipeline papers to observe trends across time and categories. In evaluating all the segmentation papers, the sample size was dichotomized into large and small cohorts with a cut-off sample size of 500, which was determined based on previously published work (Frey et al., 2019). The population used in both pipeline and segmentation articles was categorized according to the following: aging (included cognitively normal individuals), small vessel disease including stroke, diabetes, vascular disease, dementia (ex: Alzheimer Disease, Parkinson Disease), psychiatric disorders (ex: depression, bipolar disorder), or miscellaneous.

The level of scrutiny in validation and efficacy of each WMH pipeline was assessed with two separate 3-tier rating systems based on prior practice for machine learning algorithms. The validation tier system was defined as tier 1 (V1) no distinction between training and test set, (V2) a distinction between training and test set was defined, and (V3) a distinction between training and test set was defined and the technique was also applied to an external data set. A similar efficacy tier rating system was devised as (E1) only evaluated to itself with no “ground truth”, (E2) evaluated against manual or other segmentation techniques but not both, and (E3) compared to both manual and other segmentation techniques. All pipelines were rated on each scale based on our interpretation of the methods described by the authors. Beyond efficacy and validation, we examined the number of citations per article as a proxy for scientific acceptance.

## Results

### Trends of published articles

**Fig. 2:**
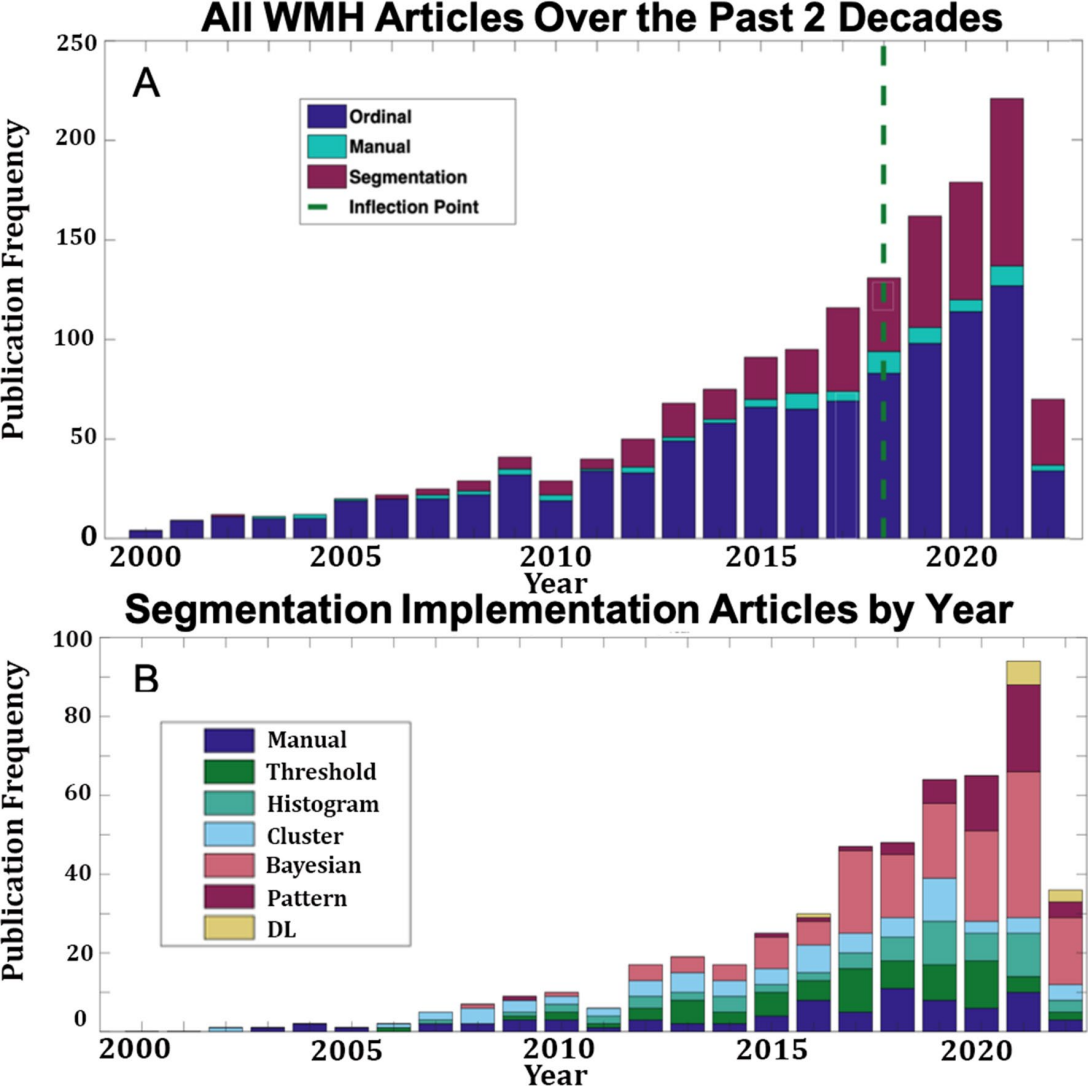
A) Frequency histogram of all evaluated WMH articles by year. B) Frequency histogram of all WMH implementation articles classified by segmentation type by year

The 1631 total articles that survived our exclusion criteria comprised 1007 visual/ordinal rating scale articles without continuous measurements of WMH burden, 118 pipeline articles, and 506 implementation articles (Fig. 1). The number of WMH publications has steadily increased over the past two decades (Fig. 2). The median number of publications per year from 2000 to 2022 was 2018. However, as the overall number of publications continues to increase, visual rating scales remained the metric of choice and far exceed the number of segmentation or manually delineated WMH publications each year for the past two decades. Manual delineation of WMH makes up the smallest portion of publications with little growth over the past two decades, whereas the proportion of non-manual automated segmentation techniques has slowly gained favor.

### Implementation publications

The 506 segmentation articles comprised 78 manual, 72 intensity thresholds, 62 histograms, 71 cluster algorithms, 160 Bayesian, 49 pattern classifiers, and 14 DL. Bayesian methods have quickly become the dominant method for WMH segmentation techniques and have grown in frequency since 2017. The utilization of DL strategies has been comparatively limited over the past two decades, with their emergence in published articles occurring predominantly after 2019 (Fig. 2B).

The sample size of the cohort might influence the amount of manual intervention necessary to oversee the segmentation process. We observed (Figure S1), very few histogram, image threshold, or pattern segmentation variants for determining WMH burden in large cohorts. Bayesian techniques, on the other hand, were more dominant in large sample sizes, though they remained the primary segmentation strategy over the past two decades regardless of cohort size.

Observing the publications across time by cohort revealed that WMH publications on aging were the most common, followed by dementia, small vessel disease (SVD), and psychiatric disorders (Figure S2). A preference for Bayesian methods was observed in the aging and dementia articles, but a shift to pattern classifiers was observed for publications involving WMH in SVD populations. There were too few publications on psychiatric disorders to suggest a trend.

### Pipeline categorization

**Fig. 3:**
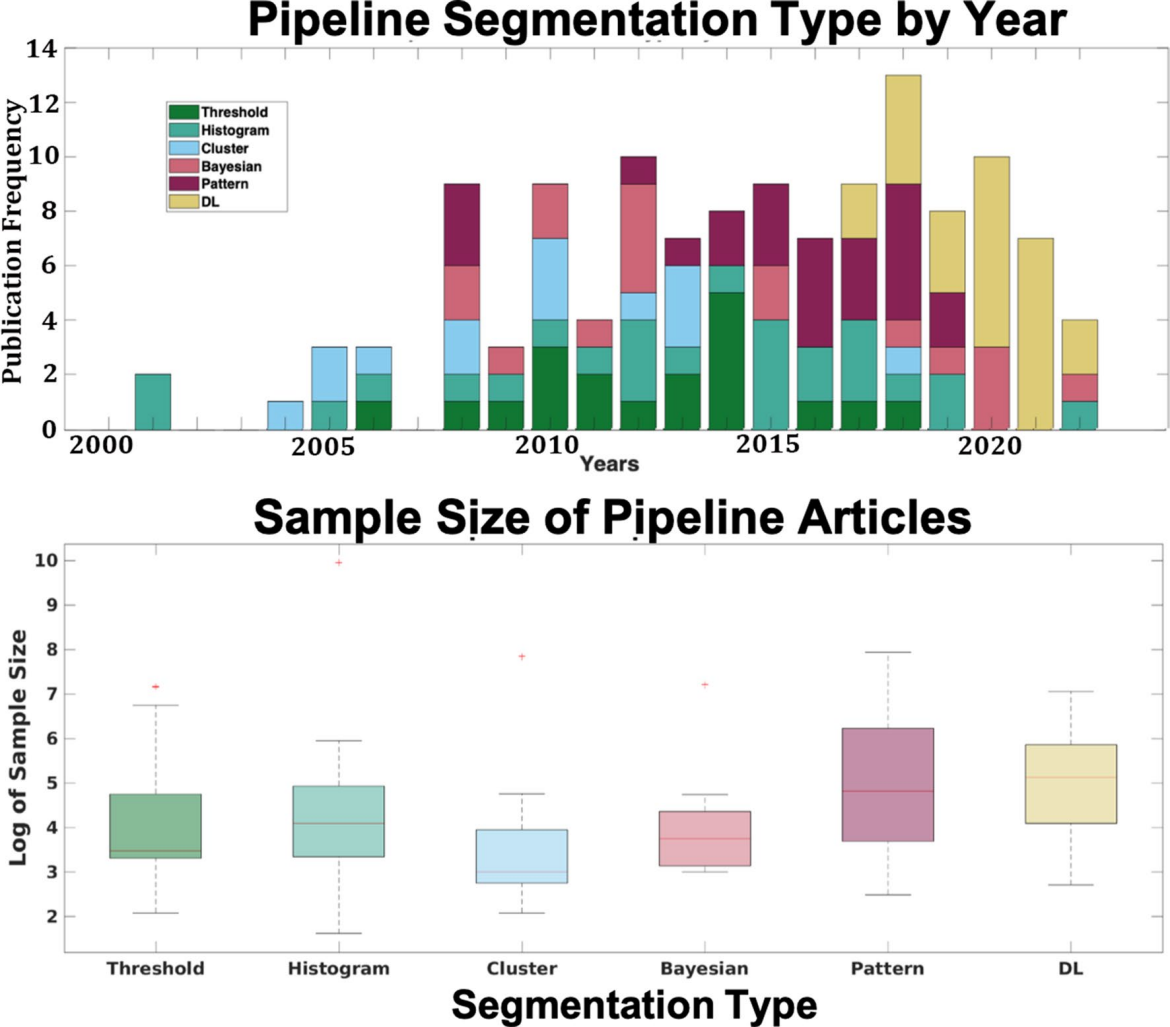
A) Frequency histogram of all WMH pipeline articles classified by segmentation type by year. B) Boxplots of all WMH pipeline article sample sizes classified by segmentation type by year. Pattern and DL articles had significantly greater sample sizes compared to all other techniques but not significantly different from each other * p < 0.05

Of the 118 pipeline articles, 18 were identified as intensity-based threshold, 25 histogram, 13 cluster algorithms, 14 Bayesian, 23 pattern classifiers, and 25 DL models. Most pipeline articles identified as cluster algorithms were some of the earliest techniques, with 70% introduced between 2004–2010 (Fig. 3A). 57% of Bayesian techniques were introduced between 2008–2012, and pattern classifiers were more frequent between 2015–2018 (67%). DL did not grow in favor until 2018 but now comprises the largest segmentation category in the past two decades. Both threshold and histogram-based techniques have the longest range across time, albeit more robustly developed before 2014 but persisted to 2019 (Fig. 3A).

### Dissection of the pipeline data

We observed larger cohorts were utilized with more technically demanding WMH pipelines. The median number of samples for both pattern classifiers and machine learning algorithms was over 100 individuals, whereas the medians of all other categories was $\le$ 60 (Fig. 3B). Mann–Whitney tests of the median revealed significantly larger sample sizes for the pattern and DL categories compared to the other segmentation types but not to each other (p < 0.05). Individually, one histogram pipeline paper was evaluated on the largest sample size (Chauhan et al., 2019), but as a category, histogram pipelines typically contained fewer participants than the pattern or DL techniques.

**Fig. 4:**
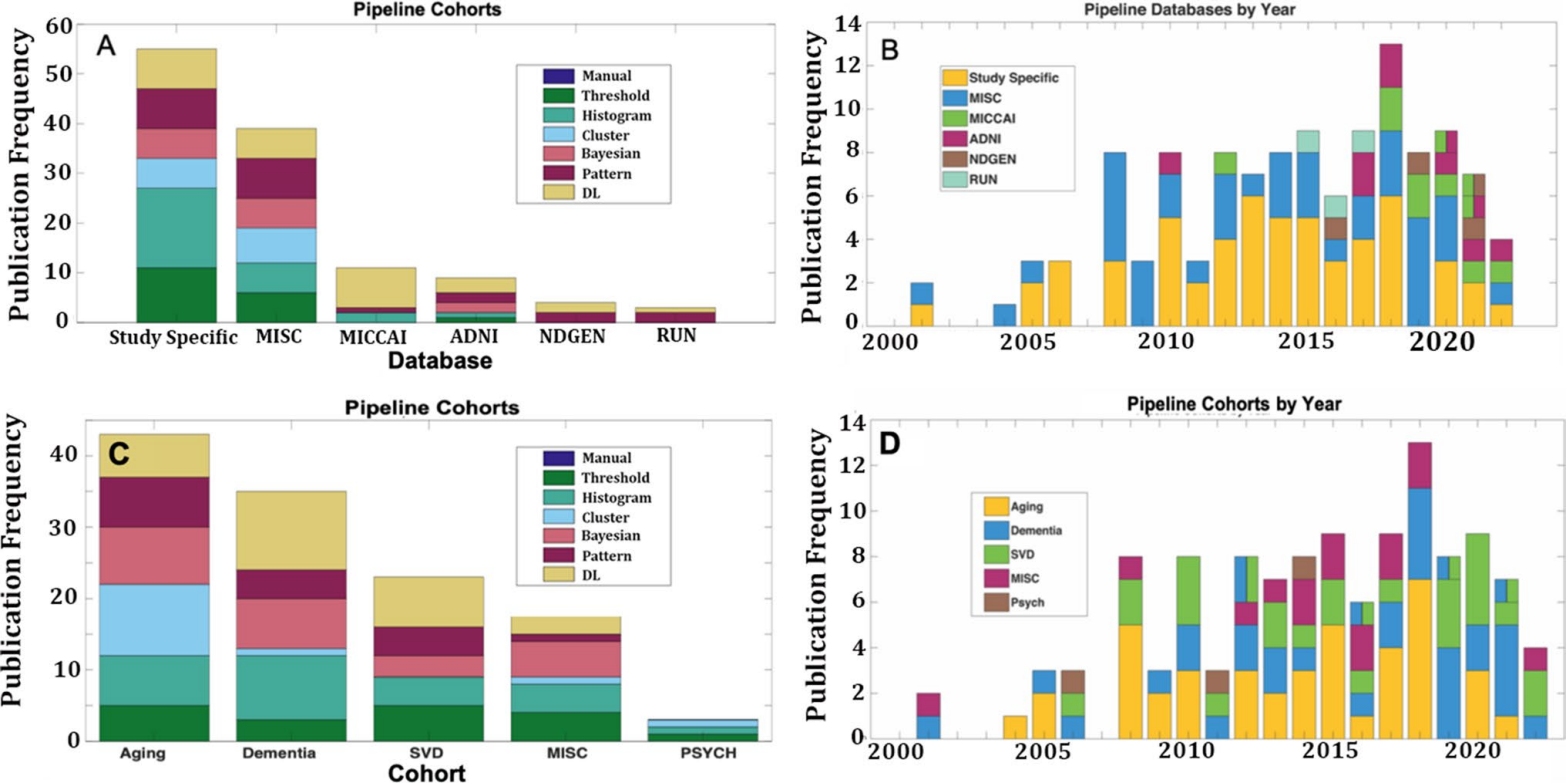
The frequency histograms show the WMH pipeline articles by cohort and/or database classified by A/C) segmentation type or B/D) by year. Only four publicly available databases were documented > 2 times across all pipeline articles. The use of publicly available databases has increased in recent years with the MICCAI database being the most preferred

Only four publicly available databases were observed in at least three pipeline papers. A greater proportion of the pipelines labeled as Bayesian, DL, and pattern classifiers utilized publicly available datasets (63%), whereas the histogram, cluster, and image segmentation algorithms (39%) were more likely to be developed on study-specific data sets (Fig. 4A). The three most utilized datasets were ADNI (Jack et al., 2010), MICCAI (Kuijf et al., 2019), and NDGEN (Griffanti et al., 2016). The ADNI cohort was the earliest founded and contained the largest variety of segmentation type categories, but the MICCAI database was more widely used. Although the second MICCAI Challenge dataset was not released to the public until after the WMH Segmentation Challenge 2017 (Deescoteaux et al., 2017), it has been used for developing more WMH pipelines than any other database (Fig. 4B). Only three pipeline papers employed a hybridized sample of individuals derived from the MICCAI Challenge and either the NDGEN or ADNI cohorts (Jansen et al., 2020; Ling et al., 2018; Sundaresan et al., 2021).

Splitting the pipeline papers by cohort revealed that aging and dementia cohorts were the most prevalent. Cognitively normal individuals have been used for developing pipelines for most of the past two decades, but a stronger focus on dementia was observed in the past 3–4 years (Fig. 4C). A growing trend for incorporating SVD and dementia cohorts into WMH pipeline development has also increased in the past 3–4 years, whereas the growth in publications for WMH in psychiatric disorders has declined (Fig. 4D). Only one pipeline article incorporated dementia, SVD, and cognitively normal individuals in assessing their WMH segmentation technique (Sundaresan et al., 2021).

### Validation and efficacy

**Fig. 5:**
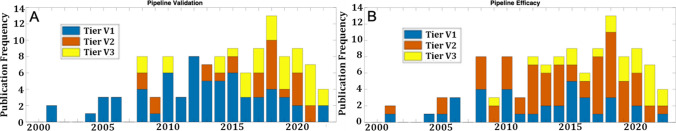
Frequency histogram of WMH pipeline articles classified by A) validation or B) efficacy by year. Validation was determined as V1) no distinction between training and test set, V2) Distinction between training and test set was defined, and V3) same as V2 but additionally applied to an external data set. Efficacy was determined as E1) only compared to itself or simulated data, E2) compared to either manual or other segmentation techniques, and E3) compared to both manual and other segmentation techniques

The level of scrutiny for assessing validation on WMH techniques has increased over the past two decades (Fig. 5). 2016 was the first year where the number of pipeline articles that validated their method against an external data set became equal to the articles which did not distinguish between the training and test sets. Similar to the validation scale, a clear shift in the efficacy tier system was observed over the past two decades (Fig. 5A). Nearly all pipeline papers compared their technique to some form of “ground truth”, whether manual or other segmentation techniques. However, the number of papers that consider both manual and other segmentation techniques for assessment has been less predominant, though this has increased in frequency in the past five years (43% percent compared to 17%) (Fig. 5B). We observed that DL methodologies had the greatest proportion of pipeline papers whose results were compared to other segmentation tools and manual rating (Image Threshold 11%; Histogram 8%; Cluster 15%; Bayesian 29%; Pattern 24%; DL 52%).

### Citation frequency

Although more DL-based pipelines were evaluated in this review, they remained the least-used techniques based on total citations. The most heavily cited WMH segmentation pipeline involved Bayesian techniques built into the SPM toolbox (Schmidt et al., 2012). This remained true after normalizing for publication years. In fact, the most cited Bayesian (SPM) and Pattern classifier (FSL) techniques (Griffanti et al., 2016) are both integrated into neuroimaging toolboxes (Figure S3).

## Discussion

Over the past two decades, several evolutions in WMH research were observed, extending to but not limited to the terminology, techniques, evaluation parameters, and pathological involvement. This comprehensive review is the first historical perspective on WMH segmentation techniques that evaluates current design trends and preferred methodologies in the field across cohort types and sizes. This review primarily focused on two sets of articles, including WMH segmentation techniques and implementation studies that apply those techniques. In general, the number of accepted manuscripts involving WMH as a primary outcome has steadily increased over the past two decades. Although the number of studies that focus on quantitative measures of WMH continues to grow, the percentage of published papers that only utilize visual rating scales remains dauntingly high across the field. Among the pipeline papers, we observed dramatic shifts from histogram and cluster-based segmentation techniques to more sophisticated DL models. Although DL-based methodologies are being developed and published at an accelerated rate compared to other techniques, the practical implementation of these techniques has thus far been much less. The most commonly used technique since 2017 continues to be Bayesian modeling and, more specifically, the SPM method (Schmidt et al., 2012). The number of citations that use the SPM method exceeded all other known techniques in popularity in the field, particularly for dementia cohorts. Whether the newer DL-based methods overtake SPM or not remains to be seen.

Indeed, one might have expected automated methods to have overtaken visual ratings. Existing standards for reporting WMH burden allow for either visual or continuous measures (Wardlaw et al., 2013) despite the known limitations of visual rating scales. However, actual comparisons between the two often show that visual scales introduce bias, inconsistencies across raters, are less reliable, lack spatial information, and perform less optimally at capturing a biological signal than continuous metrics of WMH (Heuvel et al., 2006). Employing WMH visual scales over segmentation techniques might offer an advantage in terms of feasibility. However, as the size of available data grows, this advantage is eclipsed by the computational efficiency of automated segmentation algorithms. Automated algorithms, particularly DL and supervised/semi-supervised techniques, can now also handle complex and heterogeneous datasets more effectively, offering increased precision and scalability. Nonetheless, our review shows that visual ratings remain popular. This likely reflects both an acceptance of visual ratings as well as perceived or real difficulties in implementing automated techniques. In fact, we did find more extensive implementation of segmentation techniques specifically tied to ubiquitously used neuroimaging software. Consequently, transitioning WMH studies from using visual ratings to more precise automated segmentation techniques will benefit from the inclusion of such techniques into widely used neuroimaging software, including open-source, clinical, and other commercial platforms.

In this review, we characterized a shift in strategies at the design stage to a slowly growing interest for DL in recent years. DL models are now the most popular category for pipeline development over the past two decades. This recent predilection for incorporating machine learning models, U-Nets in particular, is likely associated with the MICCAI Challenge on WMH segmentation. In 2017 the MICCAI challenge evaluated 12 techniques using identical training and test sets which revealed a strong sensitivity for delineating WMH lesion from non-lesion using U-Net models (Deescoteaux et al., 2017). Subsequent to its public release, the MICCAI dataset has been employed for training and validation in 85% of newly developed techniques.

Thus far, only a single DL algorithm has been developed and tested on multiple datasets, imposing diversity in the training and validation data, thereby enhancing the model's capacity for generalization (Sundaresan et al., 2021). We also observed that publications varied in defining the standards for determining the validity and efficacy of their proposed methodology. Standard guidelines have been established for DL algorithms to avoid over-fitting and improve reliability (e.g., nested cross-validation withhold-out stratified by age, sex, cohort, etc.). These standards are now positively influencing the field. Imposing higher validation standards on newly developed segmentation tools will lead to more reliable models and a higher degree of confidence from the medical community and, therefore, increased adoption. Independent validation studies have shown high accuracy and reliability of these newly developed techniques in large mixed datasets (Strain et al., 2024). We constructed two 3 tier rating systems that we suggest should be adapted to ensure the reliability of a given segmentation technique. Figure 5 shows that both of our metrics for validation and efficacy improved in recent years. Comparison to other accepted WMH segmentation techniques is also important, particularly since this will aid in comparing results across studies. Although only 19% (n = 13) of pipeline papers included the LST (SPM) method as a comparator, it remains widely used in WMH research and should thus be included when testing newer automated techniques.

Currently, studies that focus on dementia and aging are the two largest types of cohorts that are incorporated into training WMH pipelines and publications in the field. We observed that individuals with dementia are the leading pathological cohort studied in the past two decades, although this study did not include MS. Although SVD comprised the majority of WMH publications prior to 2019, a dramatic shift toward dementia was subsequently observed. This shift might be attributable to growing interest in the pathological role of vascular changes in AD (Hughes & Hajjar, 2021), as well as the rapidly increasing research in AD, which parallels both increases in NIH- and industry-funded studies. Findings from both pathology and spatial analyses of WMH patterns reveal AD-specific changes in WMH burden (McAleese et al., 2021),(Phuah et al., 2022). This has challenged prior models of AD to now incorporate vascular changes as an additional stage. Importantly, we also observed that developers are increasingly including individuals with dementia in training and validation datasets as a representative of abnormal brain pathology. Therefore, future WMH segmentation methods will likely be more strongly influenced by dementia pathology, which has potential implications when applied to individuals with other WMH-associated pathologies.

This review's focus is to provide a historical perspective on WMH techniques across the past two decades. This review was not designed to favor a particular pipeline or debate its advantages or disadvantages. Rather, our aim was to provide a descriptive outlook on publicly available techniques and current scientific trends within this field. We excluded articles that failed to disclose their methodology including proprietary and in-house methods. One of the focuses of this paper was to quantify and qualify the experimental designs of WMH pipeline papers rather than discuss the reliability metrics themselves, which is equally as important for determining reliability. This paper also does not meet all the requirements of a systematic review because we selected to expand our interpretation of the data. Additionally, despite our efforts to use broad terminology to identify all WMH papers published in the past two decades, we underrepresented terminology specifically used in the MS field, which could influence some of our observations. Therefore, separate reviews with even more inclusive terminology might cover the categories not included here.

Accessibility and ease of use are likely the main driving forces for why certain techniques are often selected over others. This is evident in that despite the known disadvantages of visual rating scales the publication rate of these papers has not faltered over the years. Additionally, the main utilized techniques are packaged within well known brain imaging software (SPM, FSL). This review is not designed to compare and contrast different segmentation techniques but it is apparent that there is a disconnect between pipeline development and implementation. This cycle was only modestly changed with landmark endeavors like the 2017 MICCAI challenge which inspired this slow transition to more advanced computational modeling. With the increased interest in capturing unique WMH spatial topographies that associate with varying biological sources the need for accurate and reliable techniques will continue to grow and further advance this transition. In a recent WMH technique comparison (Strain et al., 2024) from our group we observed several benefits in select techniques that employ convolutional neural networks with high reliability at both the global and regional level. As these more sophisticated techniques continue to reform and adapt in usability we predict they will become more influential in this body of work particularly for research purposes but their use in clinical utility remains to be seen.

In conclusion, we observed several trends regarding WMH assessment over the past two decades. Despite the known advantages of WMH segmentation, visual rating scales remain highly utilized. The most often utilized automated segmentation technique is the SPM segmentation method, which promotes Bayesian modeling for WMH segmentation. DL-based methods are now being increasingly developed, though their future implementation rate remains to be determined. The level of scrutiny for evaluating new WMH segmentation techniques has improved by comparing both manual and automated techniques across multiple cohorts. The MICCAI cohort is the most utilized group of individuals used for training segmentation classifiers, but more datasets should be made publicly available to avoid overfitting.

## Supplementary Information

Below is the link to the electronic supplementary material.

Supplementary file1 (XLSX 133 KB)

Supplementary file2 (DOCX 270 KB)

Supplementary file3 (DOCX 18 KB)

## Data Availability

A full list of all papers included in this review can be found in the supplemental Table 1. Fully Reproducible search strategies and queries for each database are also included in the supplemental material.
