# Supplementary material for: Evolution of white matter hyperintensity segmentation methods and implementation over the past two decades; an incomplete shift towards deep learning
Source: Brain Imaging Behav. 2024 Jul 31;18(5):1310–22. doi: 10.1007/s11682-024-00902-w (PMC11582091; doi:10.1007/s11682-024-00902-w)
Supplement: Supplementary file 2 — Supplementary file2 (DOCX 270 KB) [file 11682_2024_902_MOESM2_ESM.docx]

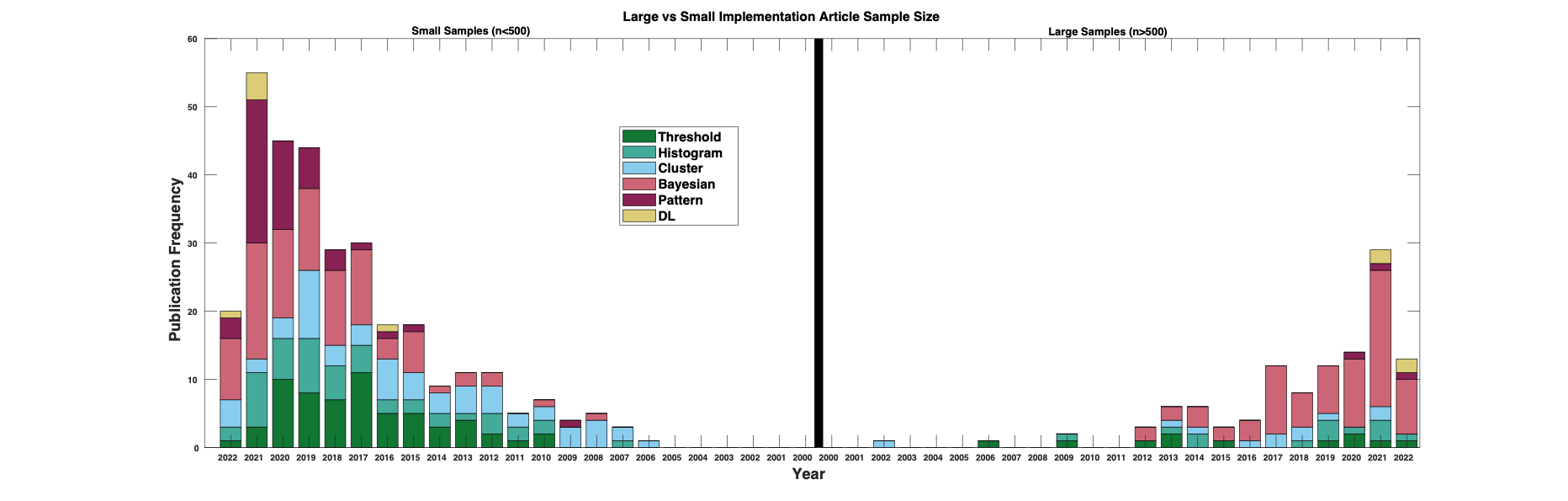


Figure S1: Frequency histogram of all WMH implementation articles classified by segmentation type by year and dichotomized by sample size. Articles that evaluated smaller sample sizes (n<500) are present on the left with the year of publication in descending order (2022-2000) and articles that evaluated large sample sizes (n>500) are present on the right with the year of publication in ascending order (2000-2022).


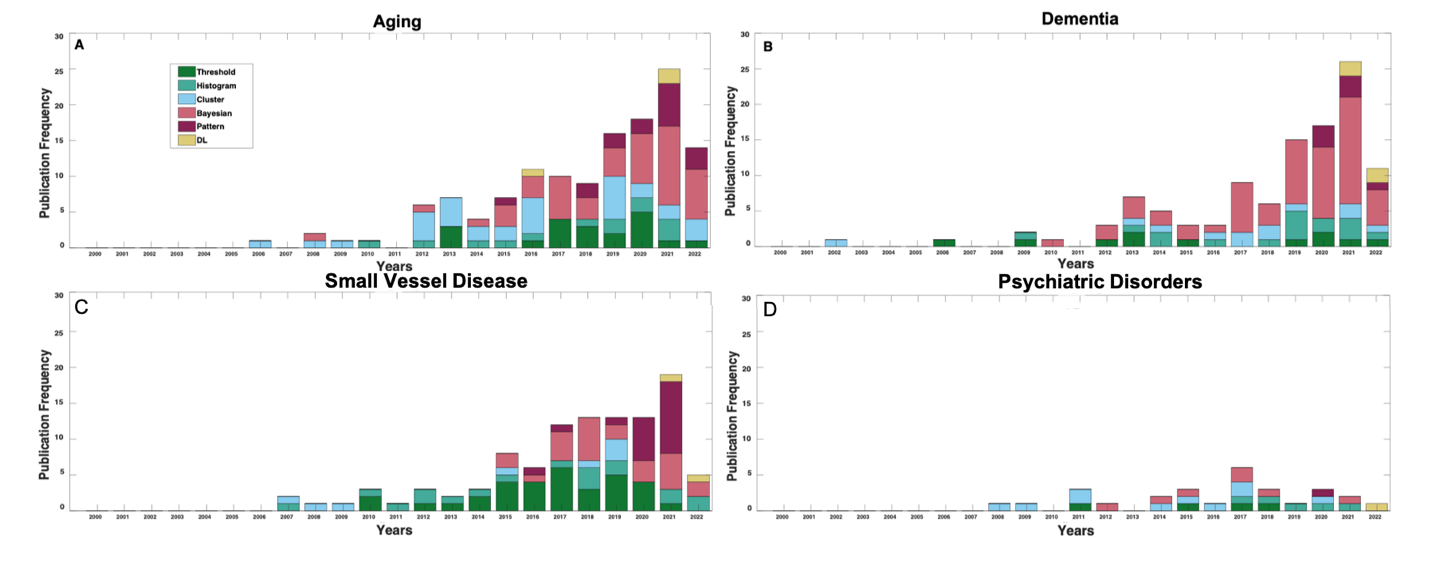


Figure S2: Frequency histogram of all WMH implementation articles classified by segmentation type by year. Each histogram represents a different etiology including: A) aging, b) dementia, c) SVD, d) psychiatric disorders.


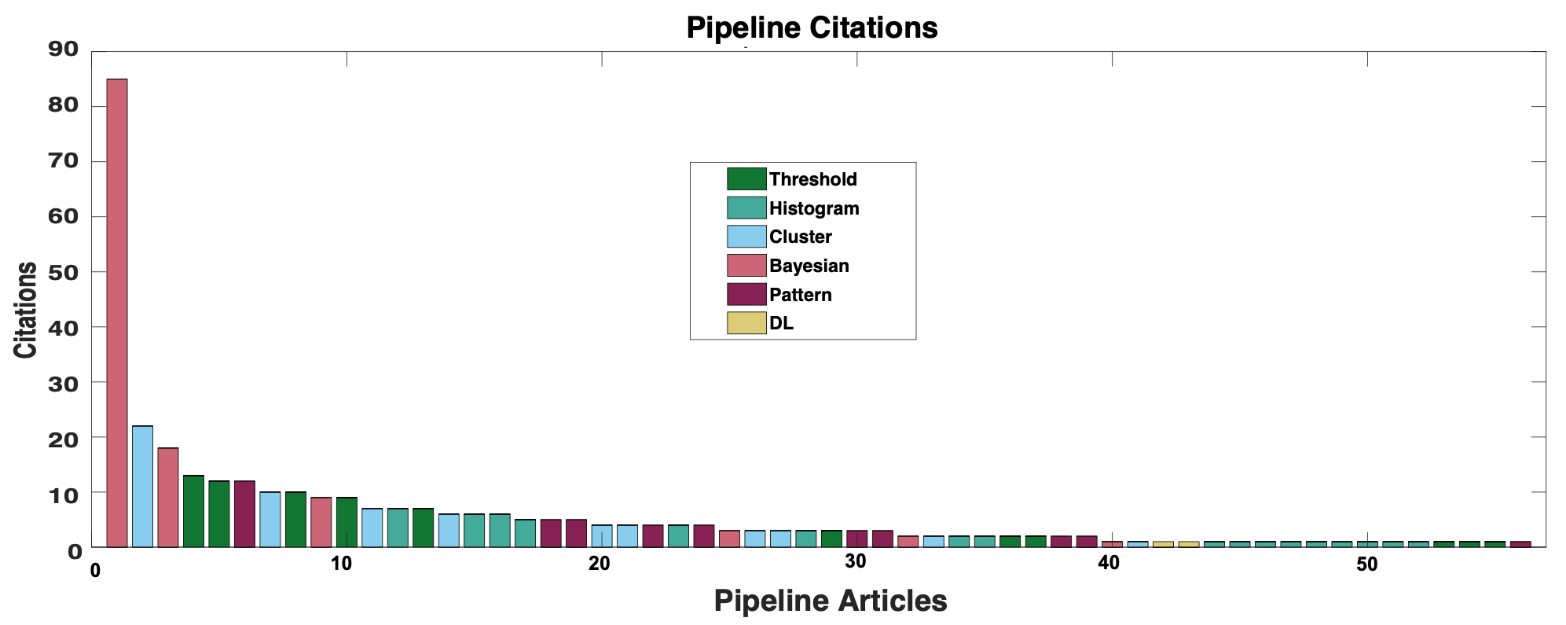


Figure S3: Frequency histogram of all WMH Pipelines cited in the literature
