# Supplementary material for: Evolution of white matter hyperintensity segmentation methods and implementation over the past two decades; an incomplete shift towards deep learning
Source: Brain Imaging Behav. 2024 Jul 31;18(5):1310–22. doi: 10.1007/s11682-024-00902-w (PMC11582091; doi:10.1007/s11682-024-00902-w)
Supplement: Supplementary file 3 — Supplementary file3 (DOCX 18 KB) [file 11682_2024_902_MOESM3_ESM.docx]

Search strategies and article screening were done using the Preferred Reporting Items for Systematic Reviews and Meta-Analyses (PRISMA) guidelines. A medical librarian (LY) created keywords and a controlled vocabulary based on variation of terms used in the literature to describe WMH according to Standards for reporting vascular changes on neuroimaging (STRIVE) 2013 guidelines(Wardlaw et al., 2013). Further, keywords were added to cover all designated segmentation strategies and, importantly, non-quantifying keywords to cover visual scales (i.e., burden). Articles were acquired across five databases, including Embase.com (n=1894), Ovid Medline (n=1579), Scopus (n=2121), Clinicaltrials.gov (n=61), and Cochrane Central Register of Controlled Trials (CENTRAL) (n=119). No specific search term was included to identify articles covering MS lesions. All search strategies were updated on November 18, 2022. Inclusion criteria included articles 1) published between 2000-2021, 2) in the English language, and 3) describing studies of adult participants; our search delivered 5,274 observations. We included pipeline articles trained on MS cohorts, but MS implementation articles were not included. 3,367 duplicate records were deleted after using the de-duplication processes described in “De-duplication of database search results for systematic reviews in EndNote,” (Bramer et al., 2016) another seven records were removed after being screened with the database tool Covidence, and 18 were manually removed resulting in a total of 2,382 unique citations included in the project library, An additional 30 articles were detected based on missing pipeline articles identified within the citations of implementation articles that survived our search criteria form which an additional 17 unique articles were included (Figure 1). Fully reproducible search strategies and queries for each database can be found in the appendix.

Abstracts and full articles were then manually screened by JS and MR based on the exclusion criteria 1) no access to the full article, 2) non-MRI imaging modalities, 3) non-adult, 4) non-human, 5) postmortem, 6) review articles that didn’t introduce a new technique, 7) articles where WMH was not designated as a primary outcome, and 8) if the methods were poorly described without citations or based on proprietary or in-house methods. 768 manuscripts were removed based on the aforementioned criteria, yielding 1631 manuscripts that met all inclusion and exclusion criteria. All papers were reviewed in Covidence and exported as spreadsheets into Matlab for figure visualization. Due to the scope of this review, we did not include these selected papers in the present reference section unless specifically mentioned; instead, a full list of all papers included in this review can be found in the supplemental Table 1.

**Fully Reproducible Copy and Paste searches:**

**Appendix**

**Full Search Strategies**

**Embase**
Date Searched: 9/17/2021

Applied Database Supplied Limits: 2000-2021

NOT ('chapter'/it OR 'conference abstract'/it OR 'conference paper'/it OR 'conference review'/it OR 'letter'/it)

Number of Results: 1,724

Search updated: 4/14/2022

Results: 1894

Full Search Strategy:

(('white matter hyperintensity'/exp OR ('hyperintensity'/exp AND ('white matter'/exp OR 'white matter lesion'/exp)) OR (('white matter' NEAR/3 hyperintensit*):ti,ab,kw) OR 'leukoaraiosis'/exp OR leukoaraiosis:ti,ab,kw OR 'leuko araiosis':ti,ab,kw) AND ('segmentation'/exp OR segmentation*:ti,ab,kw OR 'burden'/exp OR burden:ti,ab,kw OR 'load'/exp OR load:ti,ab,kw OR 'scale'/exp OR scale:ti,ab,kw) AND ('adult'/exp OR 'aged'/exp OR 'very elderly'/exp OR 'young adult'/exp OR 'middle aged'/exp OR 'frail elderly'/exp OR adult*:ti,ab,kw OR 'grown-up*':ti,ab,kw OR grownup*:ti,ab,kw OR aged:ti,ab,kw OR elderly:ti,ab,kw OR 'senior citizen*':ti,ab,kw OR senium:ti,ab,kw OR centenarian*:ti,ab,kw OR nonagenarian*:ti,ab,kw OR octogenarian*:ti,ab,kw OR 'very old':ti,ab,kw OR 'middle age*':ti,ab,kw) AND [2000-2021]/py) NOT ('chapter'/it OR 'conference abstract'/it OR 'conference paper'/it OR 'conference review'/it OR 'letter'/it)

**Ovid Medline**
Date Searched: 9/17/2021
Applied Database Supplied Limits: yr= 2000-2021
Number of Results: 1,447

Search updated: 4/14/2022

Results: 1579

Full Search Strategy:

((white matter ADJ3 hyperintensit*).ti,ab,kf. OR exp Leukoaraiosis/ OR (Leukoaraiosis OR leuko araiosis).ti,ab,kf.) AND ((segmentation* OR burden OR load OR scale).ti,ab,kf.) AND (Exp Adult/ or exp Young Adult/ OR exp Middle Aged/ OR exp Aged/ OR exp "Aged, 80 and over"/ OR exp Frail Elderly/ OR (Adult* OR grown-up* OR grownup* OR aged OR elderly OR senior citizen* OR senium OR centenarian* OR nonagenarian* OR octogenarian* OR very old OR middle age*).ti,ab,kf.)

limit to yr="2000 -Current"

**Scopus**
Date Searched: 9/17/2021
Applied Database Supplied Limits: 2000-2021
Number of Results: 1,927

Search updated: 4/14/2022

Results: 2,121

Full Search Strategy:

((TITLE-ABS-KEY(“white matter” W/3 hyperintensit*)) OR (TITLE-ABS-KEY(Leukoaraiosis OR “leuko araiosis”))) AND ((TITLE-ABS-KEY(segmentation* OR burden OR load OR scale))) AND ((TITLE-ABS-KEY(Adult* OR “grown-up*” OR grownup* OR aged OR elderly OR “senior citizen*” OR senium OR centenarian* OR nonagenarian* OR octogenarian* OR “very old” OR “middle age*”))) AND ( LIMIT-TO (PUBYEAR , 2021 ) OR LIMIT-TO ( PUBYEAR , 2020 ) OR LIMIT-TO ( PUBYEAR , 2019 ) OR LIMIT-TO ( PUBYEAR , 2018 ) OR LIMIT-TO ( PUBYEAR , 2017 ) OR LIMIT-TO ( PUBYEAR , 2016 ) OR LIMIT-TO ( PUBYEAR , 2015 ) OR LIMIT-TO ( PUBYEAR , 2014 ) OR LIMIT-TO ( PUBYEAR , 2013 ) OR LIMIT-TO ( PUBYEAR , 2012 ) OR LIMIT-TO ( PUBYEAR , 2011 ) OR LIMIT-TO ( PUBYEAR , 2010 ) OR LIMIT-TO ( PUBYEAR , 2009 ) OR LIMIT-TO ( PUBYEAR , 2008 ) OR LIMIT-TO ( PUBYEAR , 2007 ) OR LIMIT-TO ( PUBYEAR , 2006 ) OR LIMIT-TO ( PUBYEAR , 2005 ) OR LIMIT-TO ( PUBYEAR , 2004 ) OR LIMIT-TO ( PUBYEAR , 2003 ) OR LIMIT-TO ( PUBYEAR , 2002 ) OR LIMIT-TO ( PUBYEAR , 2001 ) OR LIMIT-TO ( PUBYEAR , 2000 ) ) AND ( EXCLUDE ( DOCTYPE , "re" ) OR EXCLUDE ( DOCTYPE , "cp" ) OR EXCLUDE ( DOCTYPE , "le" ) OR EXCLUDE ( DOCTYPE , "ch" ) OR EXCLUDE ( DOCTYPE , "no" ) OR EXCLUDE ( DOCTYPE , "sh" ) )

The Cochrane Library

Date Searched: 9/17/2021
Applied Database Supplied Limits: 2000-2021
Number of Results

- CENTRAL 107
- CDSR 0

Search updated: 4/14/2022

Results:

CENTRAL 119

Full Search Strategy:

((“white matter” NEAR/3 hyperintensit*):ti,ab,kw OR [mh “leukoaraiosis”] OR (Leukoaraiosis OR “leuko araiosis”):ti,ab,kw) AND ((segmentation* OR burden OR load OR scale):ti,ab,kw) AND ([mh “Adult”] or [mh “Young Adult”] OR [mh “Middle Aged”] OR [mh “Aged”] OR [mh “Aged, 80 and over”] OR [mh “Frail Elderly”] OR (Adult* OR “grown-up*” OR grownup* OR aged OR elderly OR “senior citizen*” OR senium OR centenarian* OR nonagenarian* OR octogenarian* OR “very old” OR “middle age*”):ti,ab,kw)

with Cochrane Library publication date from Jan 2000 to Dec 2021

**ClinicalTrials.gov**
Date Searched: 9/17/2021

Number of Results: 57

Search updated: 4/14/2022

Results: 61

Full Search Strategy:

(“white matter hyperintensity” OR leukoaraiosis) AND (segmentation* OR burden OR load OR scale) AND adults
